# Chemical shift extremum of ^129^Xe(*aq*) reveals details of hydrophobic solvation

**DOI:** 10.1038/s41598-018-25418-4

**Published:** 2018-05-04

**Authors:** Petri Peuravaara, Jouni Karjalainen, Jianfeng Zhu, Jiří Mareš, Perttu Lantto, Juha Vaara

**Affiliations:** 1 NMR Research Unit, P.O. Box 3000, FI-90014 University of Oulu, Oulu, Finland; 2 Research Unit of Medical Imaging, Physics, and Technology, P.O. Box 5000, FI-90014 University of Oulu, Oulu, Finland; 3 Saskatchewan Structural Sciences Center, 110 Science Place, Saskatoon, SK S7N 5C9 Canada

## Abstract

The ^129^Xe chemical shift in an aqueous solution exhibits a non-monotonic temperature dependence, featuring a maximum at 311 K. This is in contrast to most liquids, where the monotonic decrease of the shift follows that of liquid density. In particular, the shift maximum in water occurs at a higher temperature than that of the maximum density. We replicate this behaviour qualitatively via a molecular dynamics simulation and computing the ^129^Xe chemical shift for snapshots of the simulation trajectory. We also construct a semianalytical model, in which the Xe atom occupies a cavity constituted by a spherical water shell, consisting of an even distribution of solvent molecules. The temperature dependence of the shift is seen to result from a product of the decreasing local water density and an increasing term corresponding to the energetics of the Xe-H_2_O collisions. The latter moves the chemical shift maximum up in temperature, as compared to the density maximum. In water, the computed temperature of the shift maximum is found to be sensitive to both the details of the binary chemical shift function and the coordination number. This work suggests that, material parameters allowing, the maximum should be exhibited by other liquids, too.

## Introduction

Nuclear magnetic resonance (NMR) spectroscopy using ^129^Xe is commonly practiced to investigate many different materials such as gases^1^, liquids^2–4^, liquid crystals^5,6^, and porous solids^7–10^. This is because the ^129^Xe chemical shift constitutes a sensitive, inert probe of the physical properties of the host material. In particular, the aqueous solution of xenon can be viewed as a prototypical model of the solvation of hydrophobic molecules in water^11,12^. In liquid-state studies, the Xe chemical shift been found to be sensitive to the density of the solvent^13^. In particular, the xenon chemical shift tends to behave linearly as a function of temperature, reflecting the dependence of the liquid density on this parameter^13,14^. Indeed, the density of most molecular liquids is a monotonically diminishing function of temperature^15^. An analytical empirical model by Lounila *et al*.^16^ relates the local density of the solvent medium to the temperature dependence of the xenon shift in the different phases of thermotropic, uniaxial liquid crystals. This model also reproduces the linear behavior of the shift in the normal, isotropic liquid phase. However, for an aqueous solution of xenon, a non-monotonic temperature dependence of the shift is observed instead, with a maximum occurring at 311 K at the standard pressure, while the well-known temperature maximum of the water density appears at 277.13 K for normal water and 284.34 K for heavy water, D_2_O^17^. Ref.^18^ reported the occurrence of the shift maximum of ^83^Kr in aqueous solution already in 1983. The displacement of the shift maximum from the temperature of the density maximum suggests more intricate phenomena than the mere dependence of liquid density, behind the temperature dependence of the noble gas chemical shift in liquid water. The non-monotonic temperature dependence of the liquid density is a rather unique characteristic of water, which apparently renders feasible the observation of such additional features in the chemical shift. The present work implies that the underlying phenomenology may also be present in normal liquids. However, it may well occur outside the temperature range of the liquid phase.

Previously a combination of quantum-chemical electronic structure and molecular simulation studies has been used to rationalize the NMR shifts of Xe in various surroundings^19–26^. In particular, sensitivity of the xenon shift to the local liquid density, which can be understood to be represented microscopically by the coordination number *Z* of the Xe atom in the solution, has been demonstrated in computational work on Xe clusters^27^. There is a direct proportionality of the Xe shift to the number of nearest-neighbor atoms or molecules. This may be viewed as resulting from the increasing availability of vacant electronic states^27,28^, through which the negative, so-called paramagnetic shielding contributions^29^ (positive chemical shift contributions) may operate. Physically, this means more extensive deformation of the Xe electron cloud due to the intermolecular interaction. Xe chemical shift in liquid water has been previously been evaluated with a molecular dynamics (MD) simulation, *e.g*., in ref.^30^ where pairwise-additive Xe–O and Xe–H functions were used to calculate the chemical shift for the simulation snapshots. The temperature dependence of the shift was not investigated, however.

In this paper we seek to qualitatively understand and reproduce the behavior of the xenon chemical shift in aqueous solution as a function of temperature. The chemical shift is evaluated using three different approaches. In the first, we describe the system from first principles, by simulating the molecular dynamics using the AMOEBA water model^31^. For this purpose, the potential surface of the xenon-water dimer was determined quantum-chemically and the xenon-water interaction in the simulation was parameterized by fitting to this data. Trajectories obtained at different temperatures were then sampled quantum-chemically by computing the xenon nuclear shielding from instantaneous snapshots and averaging the result at each temperature. The shielding calculations were carried out at the hybrid density-functional theory (DFT) level. While the snapshot computations were performed nonrelativistically, an *a posteriori* correction to the chemical shift was added to account for relativistic effects, implemented using the zeroth-order regular approximation (ZORA)^32,33^ for a limited number of simulation snapshots. In the second, NMR ‘force field’ approach, the Xe chemical shift was approximated as a sum of preparameterized pairwise contributions from the solvent molecules, using the simulation data acquired in the first approach. This method provides an efficient way to approximate the shift in the analysis of the entire simulation trajectory without extensive QC calculations.

In the third approach, we described the system with a semianalytical model, in which the solvated Xe atom was placed in a water cavity. The cavity was modeled by a thin spherical shell, consisting of an even distribution of water molecules. The parameters of the cavity were determined by comparison to the MD simulation data wherever possible. The purpose of constructing this model was to illustrate the roles of the local solvent density (coordination number *Z*) and the Xe-water collisions, as functions of temperature, in giving rise to the shift maximum. Finally, we parameterized the cavity model entirely empirically by demanding that the experimental results are reproduced.

It is found that the temperature dependence of the ^129^Xe NMR shift in the aqueous solution of xenon can be qualitatively reproduced by both first-principles calculations and the semi-analytical cavity model with parameters fixed to MD simulation results. The cavity model can be made quantitative by fitting to experimental data. These results imply that, besides the local solvent density, also energetic aspects of solute-solvent interactions contribute to the temperature dependence of the ^129^Xe shift. The occurrence of the shift maximum is due to a combination of two opposing temperature trends: (1) the decrease of the coordination number and the chemical shift as a function of temperature and (2), larger Xe–H_2_O collision energy and closer contact of the Xe–H_2_O pairs, upon increasing *T*. Due to the very rapidly increasing Xe chemical shift function as a function of decreasing Xe–H_2_O separation, propensity for closer solute-solvent encounters produces an increasing trend of the ^129^Xe shift; one that displaces the shift maximum to a higher temperature than that of the density extremum. It may be argued that both (1) and (2) are in effect in the solvation of noble gases in all liquids, but it is the nonlinear temperature dependence of water density that renders the behavior observable in this particular solvent.

## Results

### NMR experiments

The existence of a chemical shift maximum for a noble gas in water was reported by Mazitov, Enikeev, and Ilyasov in 1987^18^. Specifically, a weak maximum was observed at approximately 45°C for ^83^Kr in an aqueous solution of krypton. Ref.^18^ also mentions briefly that a similar behavior could be observed in xenon as well.

**Figure 1:**
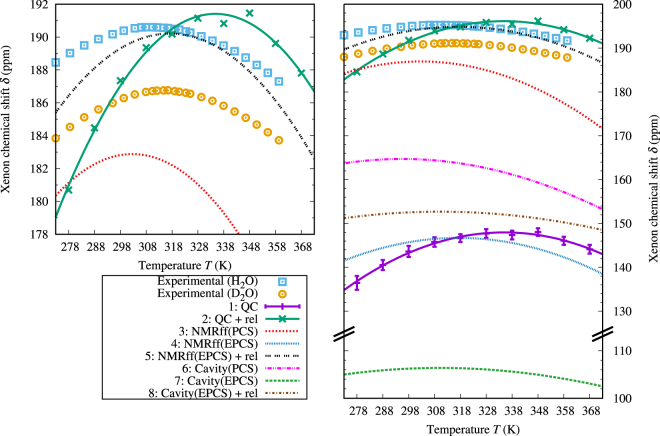
Experimental and calculated ^129^Xe chemical shift in an aqueous solution of atomic Xe as a function of temperature. NMR experimental data in normal (H_2_O) and heavy water (D_2_O), shown for both increasing and decreasing temperature series. Computational results from methods 1–8 (please refer to Table 1).

The results of our experiments on ^129^Xe(*aq*) are shown in Fig. 1, for both H_2_O and D_2_O solvents. A maximum can be observed for the Xe chemical shift at 311.13 K and 315.33 K for H_2_O and D_2_O, respectively, while the density maximum occurs at 277.13 K and 284.34 K for the two solvent isotopes, respectively^17^. The shift is approximately 4 ppm larger in H_2_O at all temperatures. The experimental temperature series for the two solvents can be successfully fitted to parabola, with the fit parameters listed in Table 1. Practically no hysteresis, *i.e*., difference between the chemical shift measured in upward and downward temperature sweeps, can be observed.

**Table 1:** Experimental and computational values of the maximum value *δ*_max_ of the ^129^Xe chemical shift in aqueous solution of atomic Xe, the temperature *T*_max_ at which it occurs and the “curvature” *a*_*δ*_ of the chemical shift, obtained with different methods.

Method^a^	*δ*_max_ (ppm)	*T*_max_ (K)^b^	${a}_{\delta }(\frac{{10}^{-3}{\rm{ppm}}}{{K}^{2}})$
Experimental (H_2_O)	190.573 ± 0.001^c^	311.13 ± 0.13^c^	1.4409 ± 0.013^c^
Experimental (D_2_O)	186.707 ± 0.014^c^	315.33 ± 0.14^c^	1.6029 ± 0.018^c^
1. QC	146.7 ± 0.2^c^	334.7 ± 1.0^c^	3.3 ± 0.2^c^
2. QC + rel^d^	191.4 ± 0.2^c^	334.7 ± 1.0^c^	3.3 ± 0.2^c^
3. NMRff(PCS)	182.9 ± 0.2^c^	302.7 ± 1.5^c^	2.9 ± 0.2^c^
4. NMRff(EPCS)	145.5 ± 0.2^c^	317.3 ± 1.0^c^	2.5 ± 0.2^c^
5. NMRff(EPCS) + rel^d^	190.2 ± 0.2^c^	317.3 ± 1.0^c^	2.5 ± 0.2^c^
6. Cavity (PCS)	162.1	295.3	1.73
7. Cavity (EPCS)	106.3	310.4	0.97
8. Cavity (EPCS) + rel^c^	151.0	310.4	0.97

The parameters *δ*_max_, *T*_max_ and *a*_*δ*_ were determined by fitting the shift vs. temperature data to a quadratic polynomial $\delta (T)={\delta }_{{\rm{\max }}}-{a}_{\delta }{(T-{T}_{{\rm{\max }}})}^{2}$.

^a^Different computational methods used. 1: Nonrelativistic QC snapshot calculations; 2: QC snapshot calculations with a posteriori relativistic correction; 3: NMR force field calculation from the MD snapshots in a pairwise-additive manner using the PCS approach; 4: NMR force field calculation with the EPCS approach; 5: NMR force field calculation with the EPCS approach with a posteriori relativistic correction; 6: The semianalytical cavity model with the PCS approach and 7: with the EPCS approach; 8: Cavity model with the EPCS approach with a posteriori relativistic correction.

^b^The density maximum of the AMOEBA water model occurs at 290 K for a classical MD simulation^38^. The experimental density maximum occurs at 277.13 K and 284.34 K for H_2_O and D_2_O, respectively^17^.

^c^The statistical error acquired by the least-squares fit.

^d^Relativistic offset.

From the perspective of classical mechanics, the difference between H_2_O and D_2_O is purely dynamic in nature, which would result in, for example, different diffusion and NMR relaxation constants. In contrast, the chemical shift is a static observable, depending only on the structure of the system. Therefore, the difference in the chemical shift between the two solvent isotopes arises fundamentally due to quantum-mechanical effects on the structure of the solution.

### Potential surface

**Figure 2:**
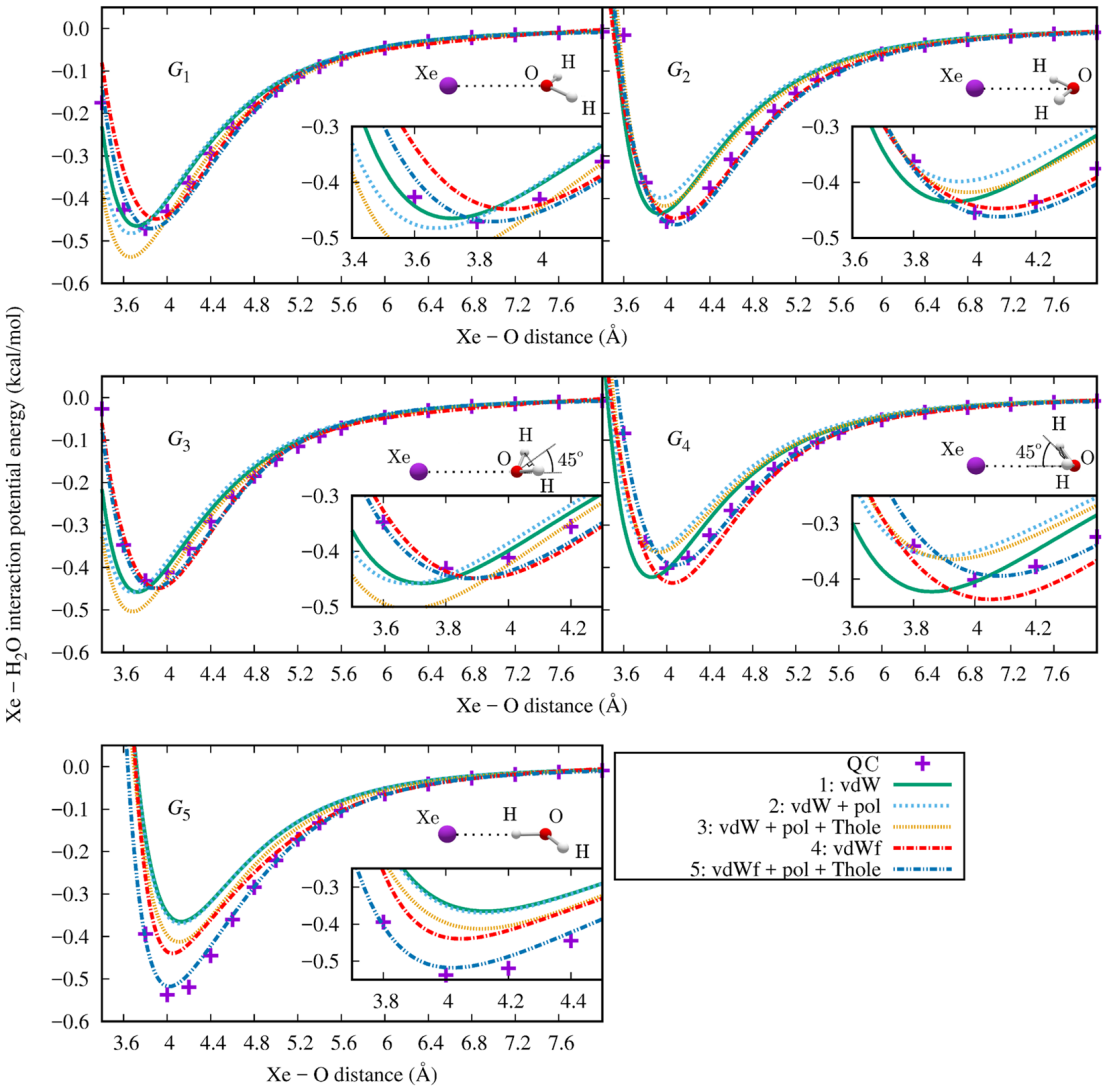
Fits of the Xe–H_2_O potential to the *ab initio* calculations (QC) with the following parameters adjusted. 1: AMOEBA van der Waals parameters; 2: van der Waals and polarizability; 3: van der Waals, polarizability, and Thole parameters; 4: coefficients *C*_*n*_ in the van der Waals potential of Eq. (S1) in the Supplementary Information; 5: polarizability, Thole factor, and the coefficients *C*_*n*_. The insets show the orientations of the Xe–H_2_O dimer for each curve family.

For both the MD simulation and the semianalytic cavity model, accurate parameterization of the Xe–H_2_O potential energy surface is necessary. In Table 2, the fitted values for the force field parameters, acquired by quantum-chemical (QC) calculation as described in the Supplementary Information, are listed along with the root-mean square (RMS) error for each fit. Figure 2 illustrates the parameterized potential energy with the different fitting schemes, along with the *ab initio* results. Parameterization of the AMOEBA van der Waals potential form can reproduce fairly reasonably the potential energy results for configurations *G*_1–4_ shown in the insets of Fig. 2, but fails to account for the depth of the potential well of *G*_5_. This property can be seen from the fit schemes 1–3 in Fig. 2. Only in the scheme where van der Waals potential is of the more general form [Eq. (S1) in the Supplementary Information] and both the polarizability and the Thole factor are optimized, is this behavior properly accounted for. The potential obtained by this arrangement (fitting scheme 5) was used in the present simulations.

**Table 2:** Parameters resulting from the different fitting schemes of the Xe–H_2_O potential, along with the RMS error for each fit. R^0^, *ε*, *α*, and *a* are the AMOEBA force field parameters for the xenon atom as described in the Supplementary Information.

Fitting scheme^a^	R^0^ (Å^3^)	ε (kcal/mol)	α (Å^3^)	a	RMS (kcal/mol)
1: vdW	4.15	2.362	4.00^b^	0.390^b^	0.934
2: vdW + pol	4.22	0.630	7.38	0.390^b^	0.902
3: vdW + pol + Thole	4.28	0.551	9.74	0.600	0.732
4: vdWf	^c^	^c^	4.00^b^	0.390^b^	0.790
5: vdWf + pol + Thole	^d^	^d^	10.50	0.422	0.543

^a^Fitted parameters, as described in Section S1.2 in the Supplementary Information; vdW = AMOEBA van der Waals potential, vdWf = modified van der Waals potential, pol = polarizability, Thole = the Thole charge distribution parameter.

^b^Values fixed to the defaults mentioned in Section S1.2 in the Supplementary Information.

^c^The coefficients used in the van der Waals function can be found in Table S1 in the Supplementary Information.

^d^The coefficients used in the van der Waals function can be found in Table S2 in the Supplementary Information.

In the AMOEBA force field, the philosophy is that the various terms and parameters retain their physical interpretations. The value obtained in the best present potential parameterization for the polarizability (volume) parameter, 10.5 Å^3^, equals the maximum value allowed for *α* and the fitting scheme 5 used in the simulation ends up exactly at this limiting value. This reflects the fact that an even better fit to the *ab initio* energies of the Xe–H_2_O dimer would have been achieved by allowing a yet higher polarizability. However, using such, higher values inhibit the polarization calculation from converging to self-consistency when short Xe–H_2_O distances occur in the MD simulation. Fortunately, even with this limitation, the best fit can reproduce the general form of the potential well in each orientation *G*_1–5_. One way of dealing with this problem would have been to do the fitting with QC data extracted from Xe(H_2_O)_*n*_ clusters, resulting in an effective Xe–H_2_O pair potential. However, the use of accurate CCSD(T) level calculations would then not have been possible due to the computational cost.

The fact that the physical interpretation of the parameters have to be abandoned this way is not unheard of with the AMOEBA model^34–37^. In ref.^34^, two of the present authors resorted to parameterizing both the polarizability and the ion charge of the Ni^2+^ ion in water to achieve a good fit to the QC results, with the value of the polarizability, similarly to the present case, clearly higher than the physical value.

### Xe chemical shift

#### Snapshot-supermolecule method

Xe NMR shielding calculations were carried out on the Xe–H_2_O dimer and several instantaneous Xe(H_2_O)_*n*_ cluster snapshots sampled from the MD simulation trajectory. The water molecules included in the calculations were such that the distance between either the oxygen or one of the hydrogen atoms from the xenon center was at most 5.5 Å, which is approximately the radius of the first solvation shell. The dependence of the Xe nuclear shielding on the cluster size in two example snapshots extracted from the simulation can be seen in Table S3 in the Supplementary Information, which shows that contributions from water molecules beyond this limit are small. Further details of the MD simulation and QC calculations of the snapshots are provided in Sections S1.4–S1.6 of the Supplementary Information.

Figure 1 shows the temperature dependence of the QC-calculated average Xe chemical shift from snapshots extracted from MD simulations. The data are listed (with additional details) in Table S4 of the Supplementary Information. The figure shows that the maximum of the chemical shift is reproduced by the QC averaging, *i.e*., AMOEBA water with nonrelativistic QC calculations of instantaneous Xe(H_2_O)_*n*_ supermolecules can reproduce the nonlinear behavior of the xenon chemical shift. The maximum shift and the temperature of the maximum for the various computational methods have been summarized in Table 1.

The magnitude of the QC chemical shift is at all temperatures approximately 40 ppm lower than the experimental results. The nonrelativistic treatment and the inaccuracy of the basis set collaboratively explain most of this difference, as discussed in the next Section. The temperature of the chemical shift maximum for the averaged snapshot calculations can be seen to be about 334.7 K, while the experimental values are 311.13 K and 315.33 K for H_2_O and D_2_O, respectively, *i.e*., the AMOEBA temperature is about 23.6 K or 19.4 K higher than the experimental one. As shown by Ren *et al*.^38^, the original AMOEBA H_2_O model has a temperature of maximum density approximately 13 K or 6 K higher than what is found experimentally for H_2_O and D_2_O, respectively. Therefore, the simulated temperature offset between the chemical shift maximum and the density maximum (45 K) has roughly the same order of magnitude as the experimental offset (34 K or 31 K). Also, the AMOEBA water density changes more rapidly with temperature than in real water^38^, which corresponds to the steeper chemical shift slope in our case. As discussed above, the difference between D_2_O and H_2_O is due to quantum-mechanical isotope effects that cannot be reproduced in a classical dynamics simulation, such as the present one. Because the quantum dynamics effects are less significant in heavy water, it is reasonable to presume that the structural parameters in our classical water simulation is closer to real D_2_O than H_2_O.

#### Relativistic corrections to the snapshot calculations

Due to the fact that Xe is a heavy element, *a posteriori* relativistic corrections were applied to the snapshot shieldings. The shifts in the relativistic ADF calculations for the twenty chosen example snapshots are listed in Table S5 of the Supplementary Information. Compared to the calculations done with the same basis sets (jcpl/TZP), the relativistic effects contribute, on the average, an additional $\mathrm{(30.3}\pm \mathrm{5.9)}$ ppm to the NR chemical shift. However, already the improvement of the basis set in the NR calculations, from the Dalton calculation with the Gaussian 27*s*25*p*21*d*4*f*/def-TZVP basis (Xe/other atoms) to the ADF calculation with the Slater-type jcpl/TZP basis, contributes another $\mathrm{(14.4}\pm \mathrm{3.0)}$ ppm, making the difference $\mathrm{(44.7}\pm \mathrm{7.0)}$ ppm in total. We applied these contributions on top of the temperature-averaged chemical shift as a systematic offset of +44.7 ppm at each temperature. As can be seen from Fig. 1, the offset brings the magnitude of the chemical shift based on QC calculations on MD snapshots to the same overall level with the experiment.

#### Preparameterized NMR force field

In the second computational approach, the ^129^Xe chemical shift was evaluated as a sum of pairwise contributions arising from the solvent molecules. Via preparameterization of the intermolecular shift function using two distinct QC approaches, the pairwise-additive chemical shift (PCS) and the effective pair chemical shift (EPCS), the simulation snapshots may be evaluated using the atomic coordinates only, and costly QC calculations of each snapshot are avoided. More details are given in Section S1.7 of the Supplementary Information.

The coefficients *δ*_*n*_ [Eq. (S20) in the Supplementary Information] used for the preparameterized chemical shift can be seen in Table S6 in the Supplementary Information. The chemical shifts calculated in a binary pairwise-additive manner, based on the PCS parameterization using the same snapshots as for the QC calculation, can be seen in Fig. 1. The chemical shift acquired using the PCS approach is approximately 40 ppm higher than from the QC calculations, while the second parameterization that takes the non-binary interactions implicitly into account, the EPCS approach, expectedly gives approximately the same magnitude as the QC calculations do. This suggests that the strictly binary pairwise-additive approximation is not sufficient to quantitatively describe the ^129^Xe chemical shift interaction in the liquid water environment. However, the fact that both the PCS and the EPCS models still produce the maximum for the chemical shift suggests that this approach, *i.e*., treating the chemical shift as a sum of pairwise contributions between xenon and water, may be used to qualitatively describe this phenomenon. It is worth noting that the shift given by the PCS approach is closer to the experimental chemical shift in magnitude. The apparent success of the PCS NMR force field is due to cancellation of two errors, neglect of the beyond-binary intermolecular interactions (included in the QC and EPCS calculations), as well as relativistic effects. Adding the relativistic offset described in above to the EPCS parameterization gives a chemical shift curve that is by far the closest to the experimental one for H_2_O (also shown in Fig. 1).

Parameterizing an effective intermolecular chemical shift function in a similar fashion has been done previously for the Xe_*n*_ clusters^27^. As in the present case, in ref.^27^ the purely binary chemical shift was found to overestimate the chemical shift, with the overestimation increasing with the cluster size.

**Figure 3:**
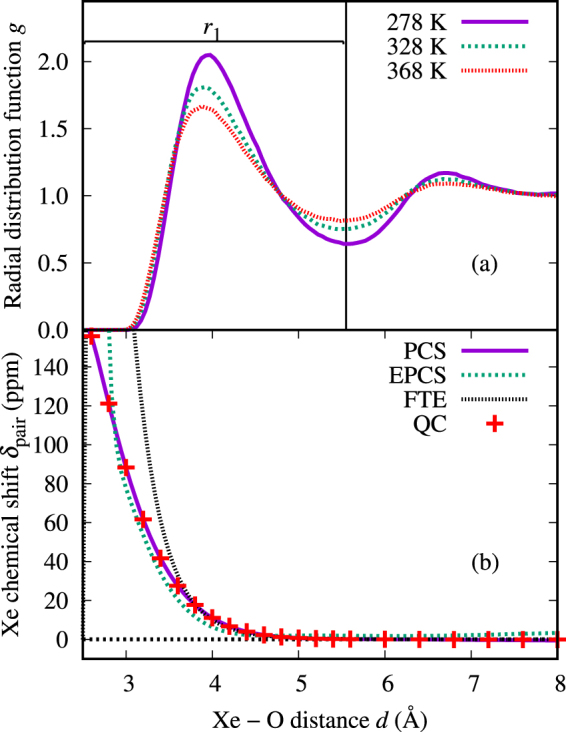
(**a**) Radial distribution functions (RDFs) *g*(*d*) for the Xe–O pairs simulated at temperatures 278 K, 328 K, and 368 K using a bin size of 0.05 Å, and (**b**) the xenon chemical shift *δ*_pair_ for the Xe–H_2_O dimer. Here, *r*_1_ corresponds to the distance to the first minimum of the RDF, *i.e*., the upper limit to the radius of the first solvation shell. In panel (**b**), the PCS and EPCS approaches (see the Supplementary Information for details) correspond to a strictly binary and effective pair chemical shift functions, respectively. FTE (fit to experiment) is the result of parameterizing the semianalytical model to experiment. QC stands for the binary first-principles data that constitute the ${\delta }_{{\rm{pair}}}^{{\rm{data}}}$ function, averaged as in Eq. (S13) in the Supplementary Information.

The difference in magnitude between the two NMR force field approaches can be understood through the shape of the pair chemical shift functions, illustrated in Fig. 3. The PCS curve has a larger value throughout the Xe–O distances appropriate to the first peak of the Xe–O radial distribution function (RDF), *i.e*., the nearest neighbors The difference ranges between 0 ppm and 8 ppm at the proximity of the RDF maximum.

As can be seen from Table 1, the temperature *T*_max_ of the chemical shift maximum for the PCS approach is approximately 14.6 K lower than for the EPCS parameterization This proves that *T*_max_ is very sensitive to the details of the pair chemical shift function, a fact that is also reflected by the cavity model, as discussed below. Even when using the EPCS parameterization, the temperature remains 17.4 K lower than in the QC snapshot results. Despite the fact that, with EPCS, both *T*_max_ and *δ*_max_ (the latter when the *a posteriori* relativistic correction is applied) are in rather good agreement with experiment, this success must be considered partially fortuitous. The difference from the parent QC data on MD snapshots, from which the EPCS parameterization was drawn, demonstrates the limits of calculating the chemical shift in a pairwise additive manner.

#### Semianalytical cavity model

The construction of the cavity model for ^129^Xe chemical shift in water solution is reported in Section S1.8 of the Supplementary Information. In the cavity model, the shift can be written as a product of two temperature-dependent factors [Eq. (S21) in the Supplementary Information],

1 $$\delta (T)={\rho }_{S}(T)K(T\mathrm{)}.$$

The first factor represents the variation of the water density as a function of the temperature *T*, demonstrated by the change of the surface density of water *ρ*_*S*_, which reflects, in turn, both the varying coordination number *Z* and the cavity radius *R*. Secondly, the factor *K*(*T*) can be interpreted as the dependence of the chemical shift on the energetics of the xenon-water collisions. The xenon chemical shift *δ*_pair_(*d*) is a very steep function of the xenon-oxygen distance *d*, as can be seen in Fig. 3. This renders *K*(*T*) very sensitive to the small-distance “onset” region of the RDF *g*(*d*), which is dictated by the Boltzmann factor corresponding to the interaction pair potential.

**Figure 4:**
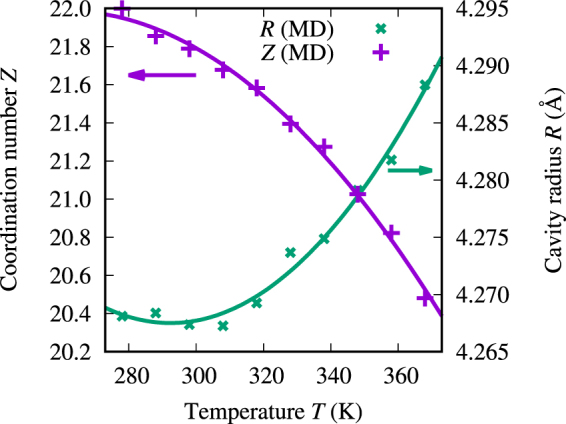
Coordination number *Z* of the xenon atom and the cavity radius *R* in the semianalytical cavity model (solid lines), as functions of temperature. MD refers to the *Z* and *R* calculated from the MD simulation trajectory as described by Eqs (S18) and (S16) in the Supplementary Information, respectively, along with fitted quadratic polynomials [Eqs (2) and (3)].

The cavity radius *R* and the coordination number *Z*, calculated from the MD trajectory, can be seen as functions of temperature in Fig. 4. The coordination number is seen to vary between 22.0 at 278 K and 20.5 at 368 K, which is consistent with the value 21.5 obtained by Schnitker and Geiger^39^ through an MD simulation with the ST2 water model^40^ at the temperature of 295 K and pressure of 1.03 atm. The corresponding changes in the simulated cavity radius with temperature are relatively small, maximally 0.02 Å. A shallow minimum for the cavity radius can be observed at 292 K, which corresponds well to the density maximum of the pure AMOEBA water at approx. 290 K^38^.

For the semianalytical cavity model, the coordination number was described with a 2^nd^-order polynomial

2 $$Z(T)={Z}_{0}-{a}_{Z}{(T-{T}_{Z}^{{\rm{\max }}})}^{2},$$

where ${Z}_{0}=21.989$, ${a}_{Z}=0.000126697\,{{\rm{K}}}^{-2}$ and ${T}_{Z}^{{\rm{\max }}}=260.56\,{\rm{K}}$. The parameters *Z*_0_, *a*_*Z*_, and ${T}_{Z}^{{\rm{\max }}}$ were acquired by least-squares fitting to the MD data. Similarly, the cavity radius was fitted to

3 $$R(T)={R}_{0}+{a}_{R}{(T-{T}_{R}^{{\rm{\min }}})}^{2},$$

where $${R}_{0}=4.2675\,\AA $$, $${a}_{R}=3.57311\cdot {10}^{-6}\,\AA /{K}^{2}$$, and $${T}_{R}^{{\rm{\min }}}=292.34\,{\rm{K}}$$.

The average chemical shift calculated using Eq. (S14) of the Supplementary Information for the PCS and EPCS parameterizations of the cavity model, is plotted in Fig. 1. For the EPCS, a curve with the relativistic offset is included as well. The PCS results are 10–20 ppm lower and EPCS results 30–40 ppm lower compared to the corresponding chemical shifts calculated pairwise-additively from the QC snapshots using the NMR force field method described above. This is probably due to the rather arbitrary manner of defining the cavity radius; particularly, choosing a definition leading to “too large” *R* leads to, due to the shape of the binary shift functions (Fig. 3b), too small shifts. Also, the curves are flat as functions of temperature compared to the QC chemical shift. This is not very surprising; as stated before, the binary shift functions have very large values at the small, relatively uncommonly occurring Xe–H_2_O separations. At the same time, with increasing temperature, the xenon atom is more often found near the cavity wall, which corresponds to those small separations. Since the cavity radius *R* is larger than the distance of the maximum of the RDF of the Xe–O distance (Fig. 3a), the changes in the total chemical shift are not as abrupt in the cavity model.

**Figure 5:**
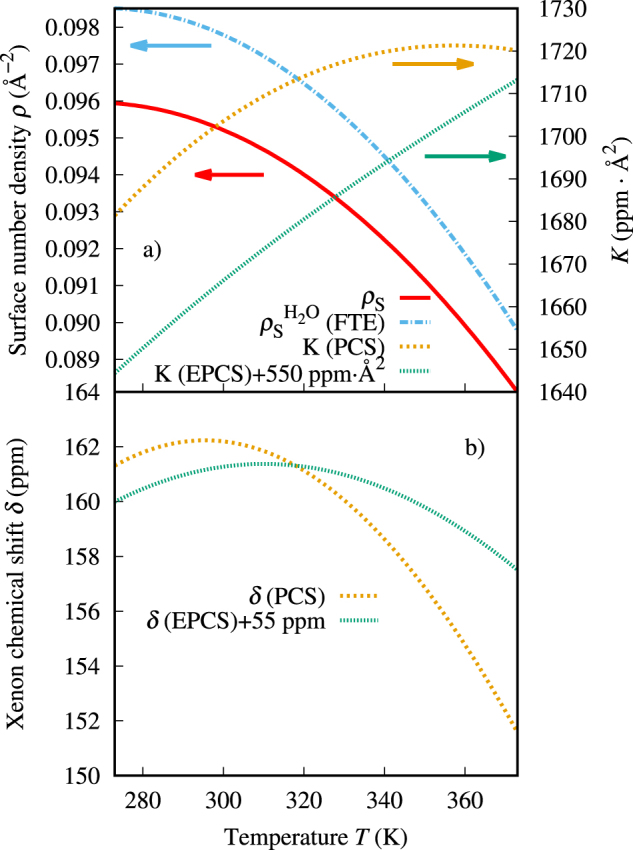
Factors in the semianalytical cavity model, *ρ*_*S*_ and *K*, defined by Eqs (S17) and (S22) in the Supplementary Information, respectively, and their product, the xenon chemical shift, as functions of temperature. PCS and EPCS refer to the strictly binary and effective pair chemical shift functions, respectively. To render the comparison easier, offsets of 550 ppm Å^2^ and 55 ppm were added to the EPCS *K* and *δ*, respectively. ${\rho }_{S}^{{{\rm{H}}}_{2}{\rm{O}}}$ (FTE) refers to the H_2_O surface density function acquired by a fit of the model to the experiment.

The surface density *ρ*_*S*_ reflects the water density contribution to the temperature dependence of the chemical shift. Therefore, it is reasonable to presume that K(*T*) is responsible for the shift in temperature of the maximum. K(*T*) and *ρ*_*S*_(*T*) are individually plotted, along with the chemical shift resulting as their product, in Fig. 5. The surface density *ρ*_*S*_ shows a behavior similar to that of the coordination number *Z*, as the variation of the cavity radius *R* with *T* is small [Fig. 4 and Eq. (S17) in the Supplementary Information]. *Z* descends nonlinearly as a function of the temperature, while the function *K*, which depends on the collision energetics and the shape of the binary chemical shift function, is an ascending function of *T*. The EPCS parameterization produces a predominantly linear *K* while for the PCS parameterization, *K* has a maximum at 357 K. In both cases, the product of the decreasing density function and the increasing “interaction function” is seen to cause the transfer of the chemical shift maximum from the maximum of the bulk solvent density to a higher temperature. A similar argument concerning the existence of the shift maximum can be put forward regarding the solvation shifts of Xe or other noble gases in any liquid. Whether the product of decreasing density and increasing collision factor leads to an observable shift maximum at a temperature at which the solution remains liquid, depends on the properties of the solvent. Here, the unique properties of water come to play a role. As a simple test, replacing the nonlinear surface density function of Fig. 5 with a linearly decaying one, also leads to the occurrence of a shift maximum, which, however, is well outside of the liquid temperature range.

The fact that the PCS *K* is an overall larger function than its EPCS counterpart, causes two differences in the resulting chemical shifts, besides their magnitude. Firstly, the temperature of the maximum corresponding to EPCS is lower. Secondly, the chemical shift curve corresponding to PCS features a sharper maximum. In the latter case, the curvature of *K* further increases the effect on the chemical shift, as well.

#### Fitting the cavity model to experiment

Up to this point we have fixed the parameters in the cavity model using the structural data originating from the MD trajectories and the chemical shift interaction data from DFT computations. This constitutes an essentially first-principles model to illuminate the physical factors underlying the observable behavior of the xenon chemical shift. In the following we, however, parameterize the cavity model to precisely reproduce the experimental temperature dependence of the Xe chemical shift.

One challenge in this process is to reproduce the difference between H_2_O and D_2_O. The binary chemical shift function and the pair potential are not expected to change between the solvent isotopes, as the electronic wave functions of the Xe-water complexes are not significantly different. However, structural differences can be expected in the local density *ρ*_*S*_ and/or the cavity radius *R*. Such isotope effects on structural properties are quantum mechanical in origin, arising from the differences of the dynamics H_2_O and D_2_O in the solution.

There are a lot of parameters in the cavity model [*δ*(*d*), *V*(*d*), *R*(*T*), and *ρ*_*S*_(*T*)], more than can be extracted from the available experimental data, *i.e*., the xenon chemical shift curves in H_2_O and D_2_O solutions. Therefore, to avoid overparameterization, the pair potential energy function *V*(*d*) was kept the same as previously, as well as the surface density function for D_2_O. As discussed above, D_2_O behaves more classically than H_2_O. Six parameters in total were used in the fit: two parameters for the binary chemical shift function *δ*(*d*), one parameter for the cavity radius *R* and three parameters for the H_2_O surface density function ${\rho }_{S}^{{{\rm{H}}}_{2}{\rm{O}}}(d)$. The binary chemical shift function was expressed as

4 $${\delta }_{{\rm{pair}}}(d)=\frac{{D}_{1}}{{d}^{{E}_{1}}}+\frac{{D}_{2}}{{d}^{{E}_{2}}},$$

where *D*_1_ and *D*_2_ are the fit parameters. Several combinations of integer exponents for *E*_1_ and *E*_2_ were tried and the combination *E*_1_ = 12 and *E*_2_ = 17 was chosen for the final fit since this produced the smallest RMS error. Because the cavity radius *R* did not vary much with temperature in the simulation [see Fig. 4], it was represented with a single, temperature-independent parameter in the fit.

The water surface density functions were expressed as quadratic polynomials

5 $${\rho }_{S}(T)={\rho }_{S}^{0}-{a}_{S}{(T-{T}_{S}^{{\rm{\max }}})}^{2},$$

where ${\rho }_{S}^{0}$, ${a}_{S}$, and ${T}_{S}^{{\rm{\max }}}$ were different for the two solvent isotopes. For D_2_O, the parameters were set to constant values, which were determined by least-squares fitting to the values acquired by Eq. (S17) in the Supplementary Information at each simulation temperature. For H_2_O, *ρ*_S_^0^, *a*_S_, and ${T}_{S}^{{\rm{\max }}}$ were free fit parameters.

**Figure 6:**
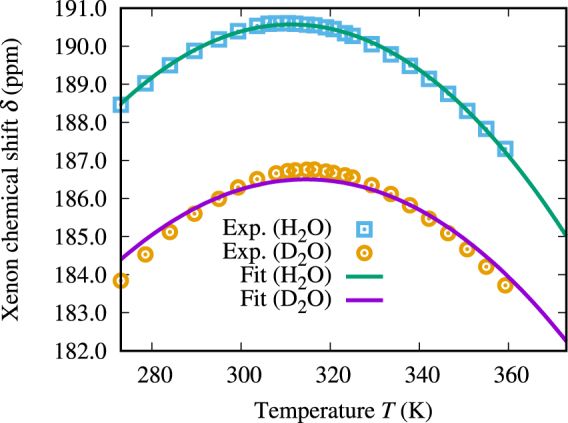
^129^Xe chemical shift in an aqueous D_2_O/H_2_O solution of atomic xenon in the semianalytical cavity model, fitted to experiment.

The fit is shown in Fig. 6 and the acquired parameters can be found in Table 3. The binary chemical shift and the H_2_O surface density functions acquired by the fit are plotted in Figs 3(b) and 5, respectively. The fit is slightly less accurate for D_2_O in general, since there were fewer parameters available. The H_2_O surface density function acquired by the fit is larger than for D_2_O, corresponding to the larger chemical shift. The chemical shift function acquired by the fit can be seen to be steeper at small Xe–O distances than the one calculated quantum chemically, which is explained by the lack of relativistic treatment in this QC shift curve. The acquired cavity radius is smaller than what was determined from MD. The cavity model chemical shift is generally smaller and broader than with the corresponding NMR force field method and, as discussed above, the smaller cavity radius in the fit accounts for both of those features.

**Table 3:** Parameters for the cavity model fit to experiment.

Isotope	R (Å)^a^	${\rho }_{S}^{0}$ (Å^−2^)	a_S_ (10^−7^ Å^−2^ K^−2^)	${T}_{S}^{{\rm{\max }}}$ (K)	D_1_ (ppm Å^12^)^a^	D_2_ (ppm Å^17^)^a^
D_2_O	4.1423	0.0959701^b^	6.99326^b^	266.975^b^	$1.85791\cdot {10}^{8}$	$-1.81437\cdot {10}^{10}$
H_2_O	4.1423	0.0985138	8.29205	270.387	$1.85791\cdot {10}^{8}$	$-1.81437\cdot {10}^{10}$

^a^Common fitted parameters for both solvent isotopes.

^b^Parameters fixed to the fit to the MD data.

Using a slightly different density function for H_2_O and D_2_O is enough to reproduce the different chemical shift curves of the two solvent isotopes. Light water has overall broader structural features than heavy water^41^, which suggests that very short Xe–H_2_O distances could be more common for H_2_O; this would explain the difference in magnitude between the surface density functions acquired by the fit.

## Discussion

NMR of dissolved, atomic xenon can be used to investigate the properties of liquids, including those of the unique solvent, water. We have qualitatively reproduced the temperature dependence of the ^129^Xe chemical shift, including the characteristic occurrence of a shift maximum, for xenon in aqueous solution using different computational methods. A combined molecular dynamics-quantum chemistry approach was realized through parameterizing the Xe–H_2_O pair potential to the polarizable AMOEBA force field and averaging the QC chemical shift in simulation snapshots. The magnitude of the solvent shift can be reproduced when relativistic effects are incorporated as an *a posteriori* correction to the shifts of the QC snapshots. The location of the maximum is 20 K or 24 K higher in temperature than the experimental ones for D_2_O and H_2_O, respectively.

Using a simple semianalytical cavity model, the temperature-dependence of the Xe chemical shift in water is found to result from an interplay of two factors. Firstly, the local density around the xenon atom displays a characteristic nonlinear temperature dependence, reflected in the cavity radius and number of nearest-neighbor water molecules. Secondly, the dynamics of the xenon-water collisions is affected by temperature: deeper impacts occur upon raising *T*. Due to the steeply increasing interaction-induced chemical shift function at small intermolecular distances, the elevated propensity to such close encounters raises the xenon shift with *T*. The location of the maximum is found to be very sensitive to the parameters of the model, which is reflected on the wide range of temperatures at which the maximum appears with different methods of calculation. The chemical shift given by the cavity model is significantly lower in magnitude compared to experiment, even with the relativistic correction. Fitting the cavity model to experiment gives both correct magnitude and location of the maximum when different functions are used for the local water density around the xenon solute for the different solvent isotopes.

The solvent density is a linearly decreasing function in most liquids other than water. However, no maximum for the chemical shift has so far been observed in them, even though the maximum in water would come about even if the local density had a completely linear temperature dependence. One explanation for the peculiarity of water is that the temperature of the maximum happens to be in the temperature range of its liquid phase.

## Methods

A 10-mm mediate-thick wall NMR tube, containing about 4 mL H_2_O or D_2_O, was connected to a vacuum line and degassed. A proper volume of Xe gas (^129^Xe 86%) was then transferred into the NMR tube. Water along with the Xe gas was then frozen to the bottom of the NMR tube by liquid N_2_. The NMR tube was then sealed with open flame. The pressure of Xe in the NMR tube was calculated to be about 5.3 and 5.4 atm for H_2_O and D_2_O samples, respectively.

^129^Xe NMR experiments were performed on a Bruker Avance III spectrometer at the field of 9.4 T (^1^H frequency 400 MHz, ^129^Xe operating frequency 110.7 MHz). A simple one pulse sequence was used, with an excitation pulse of 25° flip angle and a recycling delay of 60 s. 60 scans were accumulated for each spectrum. Variable temperature experiments were performed in the temperature range from 0 to 87 °C. The reading temperatures were calibrated with standard Bruker samples. Note that, for variable temperature experiments ^2^H lock was turned off to assure the reference of chemical shift. ^129^Xe chemical shift was referenced to 1 atm Xe gas (room temperature) at 0 ppm.

Constant temperature and pressure MD simulations were performed in a cubic simulation cell with periodic boundary conditions using the Beeman algorithm for integration (0.5 fs time step)^42,43^. Ten different temperatures were used ranging from 278 K to 368 K in 10 K intervals, each with the pressure of 1 atm. There were 255 water molecules and one xenon atom in the simulations. Potential energy for the Xe–H_2_O dimer was calculated using the Molpro quantum chemistry (QC) package^44–47^ at the coupled-cluster singles, doubles, and perturbative triples [CCSD(T)] level. Scalar relativistic effects due to the heavy xenon atom were handled with the energy-consistent effective core potential (ECP) replacing the 28 innermost electrons of this atom (ECP28MDF by Peterson *et al*.^48).^ The xenon NMR nuclear shielding tensor ***σ*** for the Xe–H_2_O dimer as well as Xe(H_2_O)_*n*_ clusters extracted from instantaneous the MD configurations were calculated using the Turbomole^49^ and Dalton^50^ codes at the nonrelativistic (NR) all-electron level. For the *a posteriori* relativistic correction, the zeroth-order regular approximation (ZORA)^32,33^ method was used to treat relativistic effects at one-component scalar-relativistic and 2-component spin–orbit levels using the Amsterdam Density Functional (ADF)^51,52^ code.

The computational and theoretical methods used in this work are described in more detail in the Supplementary Information. The datasets generated and analysed during the current study are available from the corresponding author on reasonable request.

## Electronic supplementary material

supplementary information
