# Supplementary material for: Chemical shift extremum of 129Xe(aq) reveals details of hydrophobic solvation
Source: Sci Rep. 2018 May 4;8:7023. doi: 10.1038/s41598-018-25418-4 (PMC5935698; doi:10.1038/s41598-018-25418-4)
Supplement: Supplementary file 1 — supplementary information [file 41598_2018_25418_MOESM1_ESM.pdf]

Supplementary Information for

Chemical shift extremum of ^{129}Xe(aq) reveals details of hydrophobic solvation

Petri Peuravaara, Jouni Karjalainen, Jianfeng Zhu, Jiří Mareš, Perttu Lantto, and Juha Vaara

April 12, 2018

S1 Computational and theoretical methods

S1.1 Binary potential energy

Potential energy for the $Xe - H_2O$ dimer was calculated using the Molpro quantum chemistry (QC) package[1–4] at the coupled-cluster singles, doubles, and perturbative triples [CCSD(T)] level. Scalar relativistic effects due to the heavy xenon atom were handled with the energy-consistent effective core potential (ECP) replacing the 28 innermost electrons of this atom (ECP28MDF by Peterson *et al.*[5]). The aug-cc-pV6Z[6] basis sets were used for oxygen and hydrogen. The aug-cc-pV5Z-PP valence basis set[5] was used for xenon, supplemented with three additional diffuse primitive basis functions for each of the s, p, d, f, g, and h shells to improve the description of the outskirts of the electron cloud, important for intermolecular interactions. The exponent of the first additional function was chosen to be one third of the most diffuse existing exponent in the set for that angular momentum type, the second one third of that and so on. To further improve the reproduction of intermolecular interactions, a dummy basis-set expansion center was added halfway between the xenon and the oxygen nuclei. The exponents of these primitive "mid-bond" functions were 0.9, 0.3, and 0.1 for the s and p orbitals, 0.6 and 0.2 for the d and f orbitals, and 0.35 for the g orbital. The counterpoise correction[7] was used in all calculations to compensate for the basis-set superposition error.

To account for the induction contribution to the interaction energy in the MD simulation (see Section S1.4 below), as we used a polarizable AMOEBA force field (Section S1.2), CCSD(T) with ECP for xenon with basis set aug-cc-pV5Z-PP was used for the calculation of the static dipolar polarizability of the xenon atom using the Gaussian software.[8] Using ECP as opposed to an all-electron calculation has proved to be adequate in computing the polarizability, which is pronouncedly a valence-like molecular property.[9]

The potential energy calculations were performed using 5 different arrangements of the $Xe - H_2O$ dimer, shown in Fig. S1. In all cases, the internal geometry of the water

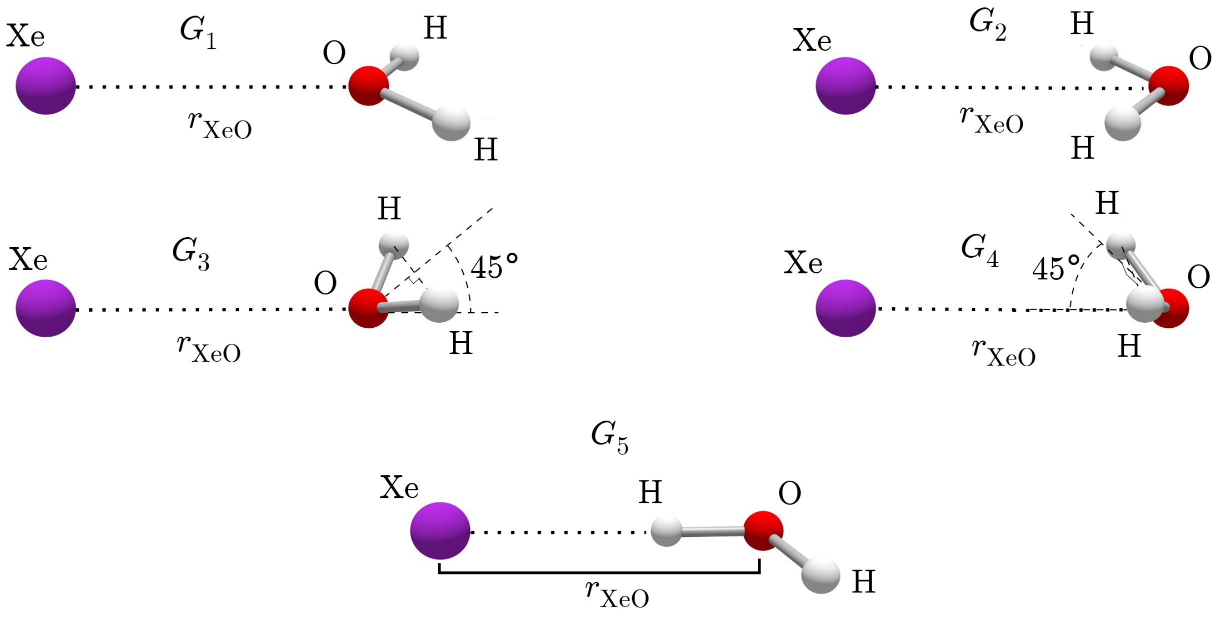

Figure S1: Orientations of the water molecule with respect to the xenon atom in calculations of the $Xe–H_2O$ potential energy function. In the G_1, G_2, and G_5 structures, all atoms are coplanar. In G_3 and G_4, the tilt angle is 45°.

molecule was fixed to the structure used in the AMOEBA parameterization of the water molecule at its equilibrium geometry, in which the bond distance $r_{\mathrm{OH}} = 0.9572\,\text{Å}$ and the bond angle $\theta = 108.5°$.[10] For each of the arrangements $G_1 - G_5$, the distance between the xenon and oxygen atoms was scanned between 3.4 Å and 5.6 Å in intervals of 0.2 Å and between 5.6 Å and 8.0 Å in intervals of 0.4 Å.

S1.2 Parameterization of the xenon-water pair potential

AMOEBA (atomic multipole-optimised energetics for biomolecular applications) is a polarizable force field for molecular dynamics simulation.[10] Unlike most MD force fields, this model is able to predict the subtle occurrence of the density maximum for liquid water, though it appears at 290 K instead of 277 K, which is the experimental value.[11] For details, see the Section S1.3.

As xenon is a neutral atom devoid of any static electric multipoles, the only interactions that are relevant for the xenon-water dimer in the AMOEBA force field are the van der Waals interaction and induction, the latter parameterized by polarizability (see Section S1.3 below). In the present work, the force field parameters for the two interactions were parameterized via least-squares fitting to QC data. Five different parameterization schemes were used:

1. Only the parameters R^0_{ii} and ε_{ii} of xenon for the AMOEBA van der Waals potential, defined by Eqs. (S2-S5), were fitted.

2. In addition to the van der Waals parameters, the xenon polarizability α_{Xe} was also an optimized parameter.

3. In addition to the van der Waals parameters and the xenon polarizability, the Thole factor a was optimized as well.

4. The van der Waals potentials for the Xe – O and Xe – H interactions were expressed, instead of the AMOEBA characteristic form of Eq. (S2), as

$$U_{\mathrm{vdW}}(r_{ij}) = \sum_n \frac{C_n}{(r_{ij})^n} \tag{S1}$$

   where the parameters C_n are optimized This form of the potential was only used to facilitate as good a fit as possible to the QC data. The xenon polarizability remains constant.

5. The polarizability and the Thole factor were again used as optimized parameters, and the van der Waals potential of the form (S1) was used.

The choice of the exponents n in the sum of Eq. (S1) was made by first using only the even exponents starting with 6, adding exponents until the fit no longer significantly improved, which occurred at $n = 12$. After that also the odd exponents starting with 7 up until 11 were included, as well.

The xenon polarizability volume of $4.000\,\text{Å}^3$ (experimental value acquired by Kumar and Meath being $4.025\,\text{Å}^3$ in Ref.[12]), obtained at the CCSD(T) level as described above, was used in the fitting schemes where the polarizability was not optimized In schemes where the Thole factor was not optimized, the AMOEBA default value of $a = 0.390$ was used.

To ensure a smooth decay of the interaction energy to zero at large intermolecular separations in the last scheme, different coefficients for the van der Waals potential were used in regions $r \leq 4.8$ Å and $r > 4.8$ Å. For the Xe – H potential, different coefficients were also used for the region $r < 2.6$ Å to ensure a fast enough ascent of the repulsive wall at small distances, described with only the terms $n = 6$ and $n = 12$. In both of these cases, two of the coefficients C_n were determined in terms of the rest by the condition that the potential and its first derivative (hence, the forces on atoms) remain continuous.

In all cases, the maximum possible values of polarizability and Thole factor were restricted to $10.5\,\text{Å}^3$ and 0.6, respectively, as the potential had a tendency not to converge to self-consistency in the MD simulation if these values were too high. Accurate reproduction of the QC energy data necessitated abandoning the one-to-one physical association of the AMOEBA potential parameters with independently observable properties, such as α, both in the present work as well as earlier.[13]

S1.3 Interactions in the AMOEBA force field

Nonbonded interactions in the AMOEBA force field consist of the van der Waals interaction as well as the electrostatic interactions between, on the one hand, permanent multipoles and due to induced multipoles, on the other hand.[10,14] The van der Waals potential of AMOEBA between two atoms i and j is written in the form

$$U_{\text{vdW}}(r_{ij}) = \varepsilon_{ij} \left(\frac{1.07}{\rho_{ij} + 0.07} \right)^7 \left(\frac{1.12}{\rho_{ij}^7 + 0.12} - 2 \right) \tag{S2}$$

where

$$\rho_{ij} = r_{ij}/R_{ij}^0, \tag{S3}$$

r_{ij} being the distance between the atoms,

$$\varepsilon_{ij} = \frac{4\varepsilon_{ii}\varepsilon_{jj}}{(\varepsilon_{ii}^{1/2} + \varepsilon_{jj}^{1/2})^2} \tag{S4}$$

and

$$R_{ij}^0 = \frac{(R_{ii}^0)^3 + (R_{jj}^0)^3}{(R_{ii}^0)^2 + (R_{jj}^0)^2}. \tag{S5}$$

R_{ii}^0 and ε_{ii} are the distance and interaction strength parameters specific to atoms of type i, and r_{ij} is the distance between atoms i and j. Permanent multipoles up to quadrupoles reside at each atomic center and constitute a multipole vector for atom i as

$$\boldsymbol{M}_i^T = [q_i, \mu_{ix}, \mu_{iy}, \mu_{iz}, Q_{ixx}, Q_{ixy}, Q_{ixz}, ..., Q_{izz}]. \tag{S6}$$

With the multipole vector, the energy of the interaction for atom i and j is

$$U_{\text{elec}}^{\text{perm}}(r_{ij}) = \boldsymbol{M}_i^T \boldsymbol{T}_{ij} \boldsymbol{M}_j \tag{S7}$$

where

$$T_{ij} = \begin{pmatrix} 1 & \frac{\partial}{\partial x_j} & \frac{\partial}{\partial y_j} & \frac{\partial}{\partial z_j} & \cdots \\ \frac{\partial}{\partial x_i} & \frac{\partial^2}{\partial x_i \partial x_j} & \frac{\partial^2}{\partial x_i \partial y_j} & \frac{\partial^2}{\partial x_i \partial z_j} & \cdots \\ \frac{\partial}{\partial y_i} & \frac{\partial^2}{\partial y_i \partial x_j} & \frac{\partial^2}{\partial y_i \partial y_j} & \frac{\partial^2}{\partial y_i \partial z_j} & \cdots \\ \frac{\partial}{\partial z_i} & \frac{\partial^2}{\partial z_i \partial x_j} & \frac{\partial^2}{\partial z_i \partial y_j} & \frac{\partial^2}{\partial z_i \partial z_j} & \cdots \\ \vdots & \vdots & \vdots & \vdots & \ddots \end{pmatrix} \left(\frac{1}{r_{ji}} \right). \tag{S8}$$

Polarization is treated via induced dipoles at the atomic centers. The induced dipole moment is defined as

$$\boldsymbol{\mu}_i^{\mathrm{ind}} = \alpha_i \boldsymbol{E}_i, \tag{S9}$$

where α_i is the static polarizability for the atom i, which is assumed isotropic, and $\boldsymbol{E}_i$ is the electric field produced by both all the static multipoles in the atoms of other molecules and the induced dipoles in all the atoms, including the ones in the same molecule. A damping mechanism introduced by Thole[15] was used to avoid the so-called polarization catastrophe" at small intramolecular distances. In this mechanism one of the point charges (it does not matter which[15]) in each pairwise interaction of induced dipoles is replaced by a charge distribution that in AMOEBA has the form

$$\rho = \frac{3a}{4\pi} \exp(-au^3), \tag{S10}$$

where $u = R_{ij}/(\alpha_i \alpha_j)^{1/6}$ and a is the dimensionless Thole factor. Because of this, the elements of the matrix T_{ij} are modified by a factor which depends on the interaction type, given by Thole[15] for the point charge, and Ren *et al.*[10] for the higher multipoles.

S1.4 Molecular dynamics simulation of Xe in water

The MD simulations were performed using the AMOEBA model with the Tinker molecular modeling package,[10,16] along with the fitted potential parameters for the xenon-water interactions, as described above. For the water solvent itself, the default AMOEBA parameters were used. The time step for the simulations was 0.5 fs and the total simulation time (stabilization excluded) was 3 ns for each temperature. The cut-off distance for all the non-bonded interactions was selected at 9.0 Å and the Ewald summation technique[17] was used for the long-range electrostatic interactions. The thermostat algorithm applied during the production simulations was extended Nosé-Hoover.[18] Prior to gathering the production part of the trajectory, the system was stabilized using the Bussi-Parrinello thermostat algorithm[19] for a period of 18 ps at each temperature. The Berendsen barostat algorithm[20,21] with the coupling time of 2.0 ps was applied in both equilibration and production phases. Snapshots for calculating the ^{129}Xe NMR chemical shift were sampled every 2125 steps, corresponding to 1.0625 ps.

S1.5 Nonrelativistic shift calculations

The xenon NMR nuclear shielding tensor $\boldsymbol{\sigma}$ for the $Xe - H_2O$ dimer as well as $Xe(H_2O)_n$ clusters extracted from instantaneous the MD configurations were calculated using the Turbomole[22] and Dalton[23] codes at the nonrelativistic (NR) all-electron level. $\boldsymbol{\sigma}$ was determined using the gauge-including atomic orbital (GIAO) hybrid DFT method with the BHandHLYP functional,[24] which has proven to be a successful DFT approach for Xe shielding in previous studies.[25–27] The basis sets for oxygen and hydrogen were of the def-TZVP[28] type, whereas for xenon, an uncontracted $(27s25p21d4f)$ basis set by Hanni[29] was used, as modified by Lantto to have four f-type basis functions instead of one.[30] The basis set was originally developed for the purpose of determining the magnetic properties of ^{129}Xe in a van der Waals complex with the rubidium atom.[31] For the exponents, see Supporting Information of Ref. 30.

The chemical shift for the xenon atom was calculated using the shielding constant $\sigma_{\text{iso}} = \frac{1}{3}\text{Tr}\,\boldsymbol{\sigma}$ as

$$\delta = \frac{\sigma_{\text{ref}} - \sigma_{\text{iso}}}{1 - \sigma_{\text{ref}}}, \tag{S11}$$

where σ_{ref} is the shielding constant of the free xenon atom.

S1.6 Relativistic shift calculations

Because of computational cost, the calculations of the $Xe(H_2O)_n$ snapshot clusters, described in Section S1.5, were carried out at the nonrelativistic (NR) level. In order to estimate the magnitude of the relativistic effects on the Xe shift, we calculated the shift relativistically for two individual snapshots from each of the simulation temperatures. In each case, one snapshot was taken randomly from the simulation trajectory, while the other corresponded to the lowest-energy configuration among those snapshots. The zeroth-order regular approximation (ZORA)[32,33] method was used to treat relativistic effects at one-component scalar-relativistic (SR) and 2-component spin–orbit (SO) levels using the Amsterdam Density Functional (ADF)[34,35] code. The ADF calculations of the NMR shielding tensors[36,37] employed the finite Gaussian nuclear model[38] with all-electron, Slater-type jcpl/TZP basis sets[39] for Xe/other atoms.

S1.7 NMR force field

The total Xe chemical shift was approximated as a sum of pairwise contributions from the surrounding water molecules. All of the available simulation snapshots, which were saved every tenth simulation step, were used to calculate the average shift at all different temperatures. The binary chemical shift function was of the form

$$\delta_{\text{pair}}(d) = \sum_n \delta_n / d^n, \tag{S12}$$

where the coefficients δ_n were determined in two different ways. In the first case, a strictly binary chemical shift was fitted to the QC results for one xenon atom and one water molecule. This case is referred to as the pairwise-additive chemical shift (PCS). When compared to the QC results for the $Xe(H_2O)_n$ MD snapshot clusters, the PCS approach can be seen to overestimate the magnitude of the chemical shift. To improve upon the pairwise treatment of the chemical shift, in the second approach to determine the coefficients δ_n, the xenon shift was interpreted as a sum of pairwise contributions of the water molecules in the $Xe(H_2O)_n$ cluster snapshots. The parameters for this interaction were least-squares fitted to the QC shielding calculations of all the clusters. This case is referred to as the effective pair chemical shift (EPCS).

For the purpose of determining the coefficients δ_n in the PCS approach, one needs information on the orientations of the water molecules in the first solvation shell of the xenon atom. The distribution of angles between the water dipole vector and the Xe – O direction was obtained from the MD simulation trajectory. The water molecules included in the calculations were such that the distance d was maximally r_1, as defined in Section S1.8 below. The plot for the distribution at three selected temperatures can be found in Fig. S2. The deviation from an even distribution is not large enough to favor a specific orientation over the others. Therefore, the fitting data used for the PCS approach

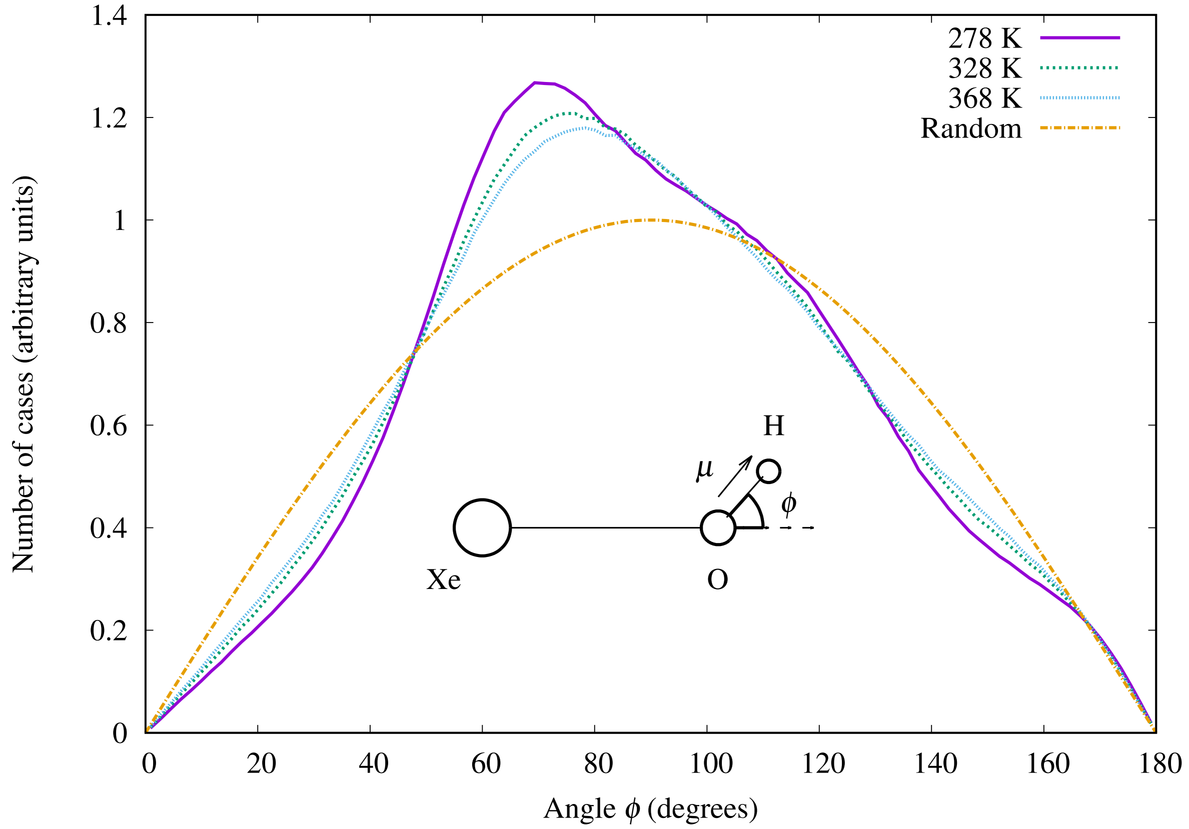

Figure S2: The distribution of the angles between the water dipole vector and the Xe–O direction calculated from the MD simulation trajectory for temperatures 278 K, 328 K, and 368 K with a bin size of 1.8°. A completely random distribution is also included for comparison.

was calculated as

$$\delta_{\text{pair}}^{\text{data}}(d) = \frac{\delta_1(d) + \delta_2(d) + \frac{1}{2}[\delta_{31}(d) + \delta_{32}(d)]}{3}, \tag{S13}$$

$\delta_i(d)$ corresponding to the configuration P_i in Fig. S3. As previously, the water bond length and angle were fixed at the values used in the AMOEBA force field. The configurations $P_1, P_2, ...$ are such that the Xe − O direction is along one of the principal axes of the moment of inertia tensor of the water molecule. Different weights used for the two geometries P_{31} and P_{32} are due to the fact that they correspond to the same principal axis from two opposite directions of approach, and the chemical shift value for that principal axis is taken to be their average. The Xe − O distances ranged from 2.6 Å to 5.6 Å at intervals of 0.2 Å and from 5.6 Å to 8.0 Å at intervals of 0.4 Å.

S1.8 Semianalytical model of the water cavity

To analyze qualitatively the ^{129}Xe chemical shift in the xenon-water system, we constructed a semianalytical approach, in which the interaction between the xenon atom and the water cavity was modeled by surrounding the solute with a thin spherical shell of water molecules, with the radius R and a continuous distribution of solvent molecules with the surface number density ρ_S. Both the interaction potential and the Xe chemical shift in the model were approximated to be pairwise additive for the interactions between the

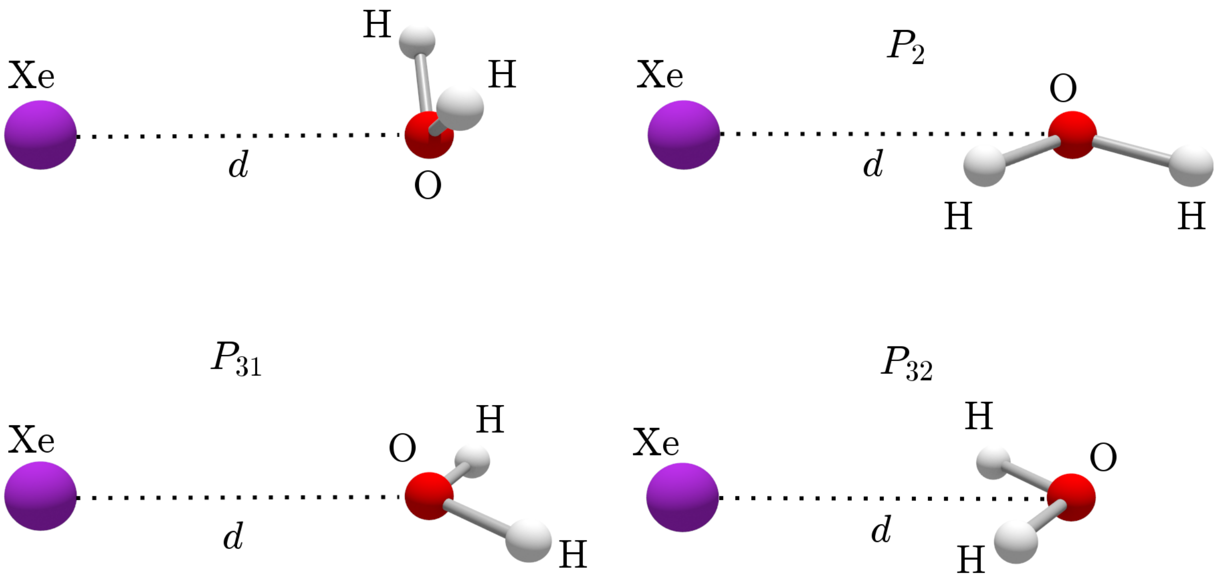

Figure S3: Orientations of water molecules with respect to the xenon atom in calculation of the pairwise potential for the semianalytical cavity model and chemical shift for the NMR force field calculations and semianalytical cavity model.

Xe atom and the solvent molecules. The xenon solute can be considered "rattling" inside the cavity with an instantaneous displacement r from the center, illustrated in Fig. S4.

The expectation value of the chemical shift inside the cavity can be written as

$$\delta(T) = \int_0^{r_{\max}} \delta_{\text{cav}}(r,T)p(r,T)\,\mathrm{d}r, \tag{S14}$$

where

$$p(r,T) = \frac{e^{-V(r,T)/kT} r^2}{\int_0^{r_{\max}} e^{-V(r,T)/kT} r^2\,\mathrm{d}r} \tag{S15}$$

is the probability density of finding the xenon atom inside the cavity at the displacement r. $V(r,T)$ and $\delta_{\text{cav}}(r,T)$ are the potential energy and chemical shift functions of the xenon atom. The value $r_{\max} = 1.5$ Å was used as the upper integration limit since the probability density $p(r,T)$ is in all cases sufficiently close to zero at that point. The temperature dependence of the $V(r,T)$ and $\delta_{\text{cav}}(r,T)$ functions arises from the fact that they involve a T-dependent structural factor (*vide infra*).

The radius R represents the size of the first solvation shell and was determined from the radial pair distribution function (RDF) of the Xe – O distances in the simulation (computed from the MD trajectories with the VMD analysis software[40]) by calculating the expectation value of the Xe – O distance up to the first minimum of the RDF,

$$R(T) = \frac{\int_0^{r_1} dg(d,T)\,\mathrm{d}d}{\int_0^{r_1} g(d,T)\,\mathrm{d}d}, \tag{S16}$$

where $r_1 = 5.55$ Å and $g(d,T)$ is the RDF. The distance r_1 was kept constant because the RDFs, seen in Fig. 3 in the main article for three selected temperatures, show very little change in the location of the first minimum as a function of temperature. The surface density was defined as

$$\rho_s(T) = \frac{Z(T)}{4\pi R(T)^2} \tag{S17}$$

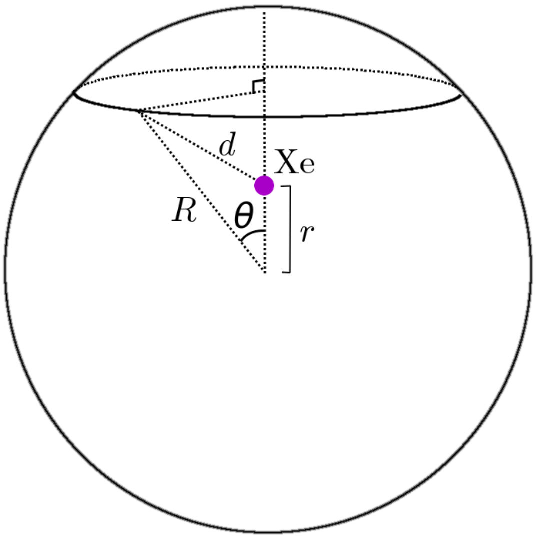

Figure S4: Illustration of the semianalytical water cavity model. The displacement of xenon from the center is r while its distance to the shell wall element at a given polar angle θ is d. The radius of the sphere is R.

where the coordination number Z is also calculated from the RDF as

$$Z(T) = 4\pi\rho(T) \int_0^{r_1} g(d,T) d^2 \, \mathrm{d}d \qquad \text{(S18)}$$

where $\rho(T) = N/V(T) = 255/L(T)^3$ is the average number density of water molecules in the simulation box with the average side length $L(T)$.

The effective potential energy of the Xe atom in the cavity model can be written as

$$V(r,T) = \rho_S(T) \sum_{n=6}^{14} V_n I_n(r,R) \qquad \text{(S19)}$$

and the chemical shift as

$$\delta_{\text{cav}}(r,T) = \rho_S(T) \sum_{n=6}^{14} \delta_n I_n(r,R), \qquad \text{(S20)}$$

where the coefficients V_n and δ_n are fitted to the intermolecular potential energy function and the binary Xe chemical shift in the interacting $\mathrm{Xe} - \mathrm{H_2O}$ pair, respectively. $I_n(r,R)$ is an analytical function resulting from the integration of the interaction between the Xe atom with an element of the cavity wall, over all the wall elements. The interaction of the xenon-water dimer is a sum of terms of the form $1/d^n$, with the corresponding number n. For details, see Section S1.9 below. With these choices for the potential and chemical shift functions, the total temperature-dependent chemical shift of the cavity model [Eq. (S14)] becomes

$$\delta(T) = \rho_S(T) K(T), \qquad \text{(S21)}$$

where

$$K(T) = \sum_{n=6}^{14} \delta_n \int_0^{r_{\max}} I_n(r) p(r,T) \, \mathrm{d}r. \qquad \text{(S22)}$$

Similarly to what was described in Section S1.7 for the PCS fitting of the chemical shift, the potential energy data used for the coefficients V_n in Eq. (S28) (*vide infra*) was calculated as

$$V_{\text{pair}}^{\text{data}}(d) = \frac{V_1(d) + V_2(d) + \frac{1}{2}[V_{31}(d) + V_{32}(d)]}{3} \tag{S23}$$

where $V_i(d)$ corresponds to configuration P_i in Fig. S3, calculated using the previously parameterized AMOEBA potential. The calculated Xe – O distances ranged from 2.0 Å to 7.0 Å with intervals of 0.1 Å, for each orientation.

S1.9 Details of the cavity model

Let A_{pair} be a pairwise additive property associated with the Xe – H_2O interaction and parameterized in the form

$$A_{\text{pair}}(d) = \sum_n \frac{A_n}{d^n}, \tag{S24}$$

where d is the distance between the xenon atom and the cavity wall. As the distance between the xenon and the surface is (Figure S4)

$$d = \sqrt{R^2 + r^2 - 2rR\cos\theta}, \tag{S25}$$

the total value of the quantity can be written as

$$A_{\text{total}}(r) = \rho_S \sum_n A_n R^2 \int_0^{2\pi} \mathrm{d}\phi \int_0^{\pi} \frac{\sin\theta\,\mathrm{d}\theta}{(R^2 + r^2 - 2rR\cos\theta)^{n/2}} \equiv \rho_S \sum_n A_n I_n(r), \tag{S26}$$

where

$$\begin{aligned} I_n(r) &= R^2 \int_0^{2\pi} \mathrm{d}\phi \int_0^{\pi} \frac{\sin\theta\,\mathrm{d}\theta}{(R^2 + r^2 - 2rR\cos\theta)^{n/2}} \\ &= 2\pi \frac{R}{r} \frac{(r-R)^2(r+R)^n - (R-r)^n(r+R)^2}{(n-2)(R^2-r^2)^n}, \end{aligned} \tag{S27}$$

provided that $n > 2$.

Both the pair potential and pair chemical shift can be conveniently fitted to be the form (S24), *i.e.*

$$V_{\text{pair}}(d) = \sum_n V_n/d^n \tag{S28}$$

and

$$\delta_{\text{pair}}(d) = \sum_n \delta_n/d^n, \tag{S29}$$

from which the coefficients V_n and δ_n follow.

S2 Results

S2.1 Potential surface

Table S1: Coefficients C_n (kcal/mol · Å^n) for the $Xe - H_2O$ potential energy function obtained for the van der Waals interaction in the fitting scheme 4.

n	Xe-O	Xe-H
6	1310689.71	-218214.476
7	-35429966.6	4937545.52
8	396196927	-46027048.0
9	-2340855590	225206026
10	7690972840	-609301598
11	-13309291700	864388166
12	9477377670	-502548480

Table S2: Coefficients C_n (kcal/mol · Å^n) for the $Xe - H_2O$ potential energy function obtained for the van der Waals interaction in the fitting scheme 5.

	Xe-O		Xe-H		
n	$r < 4.8$ Å	$r > 4.8$ Å	$r < 2.6$ Å	2.6 Å $< r <$ 4.8 Å	$r > 4.8$ Å
6	801555.018	4876.79102	24.7660805302	-222292.216	-696.652497
7	-20773611.3	-	-	4966925.83	-
8	225279395	-275293.311	-	-45687194.5	452.836189
9	-1302803650	-	-	220688180	-
10	4218217740	4079268.19	-	-590226520	102193.118
11	-7227315890	-	-	829217086	-
12	5112459130	-16232604.1	125052.162836	-478390319	-340001.857

S2.2 Xe chemical shift

Table S3: ^{129}Xe chemical shift as a function of the maximum distance allowed between the Xe atom and the water molecules for two simulation snapshots.

	Number of molecules		Chemical shift (ppm)	
Maximum distance (Å)	Snapshot 1	Snapshot 2	Snapshot 1	Snapshot 2
3.9	7	6	83.04	72.69
4.3	11	15	93.28	91.77
4.7	18	19	100.25	96.01
5.5	24	26	102.20	95.88
6.7	51	48	103.29	95.17
7.4	68	64	104.49	96.07

Table S4: Number of simulation snapshots and the computed average ^{129}Xe chemical shifts [nonrelativistic DFT(BHandHLYP) calculation with the def-TZVP basis set for O and H and $27s25p21d4f$ for Xe] at several temperatures, along with the standard error of mean (SEM), statistical inefficiency s,[41] and the estimated error $\Delta\delta$. The error was calculated as $\Delta\delta = \sqrt{s} \cdot \Delta\delta^{\mathrm{SEM}}$.[41]

T (K)	N	δ (ppm)	$\Delta\delta^{\mathrm{SEM}}$ (ppm)	s	$\Delta\delta$ (ppm)
278	2706	136.0	0.7	4.5	1.5
288	2685	139.8	0.8	2.2	1.1
298	2687	142.7	0.8	2.5	1.2
308	2687	144.7	0.8	1.8	1.1
318	2686	145.5	0.8	1.2	0.9
328	2686	146.5	0.9	1.3	1.0
338	2687	146.1	0.9	1.3	1.0
348	2686	146.8	0.9	1.0	0.9
358	2686	144.9	0.9	1.0	0.9
368	2684	143.1	0.9	1.2	1.0

Table S5: Calculated ^{129}Xe chemical shift (in ppm) using relativistic (SR and ZORA) and nonrelativistic (NR) methods on the ADF program for twenty selected MD snapshots throughout the simulation of the aqueous solution of atomic xenon at different temperatures. T represents the temperature of the trajectory from which the snapshot was taken, either the configuration of minimum energy (Min. en.) or randomly chosen (Rand.). The average value and the standard deviation (STD) are also represented for the chemical shifts at each level of theory.

T	Snapshot	NR-TM[a]	NR-ADF[b]	SR-ZO[c]	SO-ZO[d]	Δ_{NR}[e]	Δ_{rel}[f]	Δ_{NR+rel}[g]
278	Min. en.	76.8	87.8	102.6	109.2	11.0	21.5	32.5
288	Min. en.	137.4	155.6	177.4	185.3	18.2	29.7	47.9
298	Min. en.	146.7	162.2	182.9	190.5	15.5	28.3	43.8
308	Min. en.	173.3	182.6	211.5	222.7	9.3	40.1	49.5
318	Min. en.	141.3	158.6	179.7	188.4	17.3	29.8	47.1
328	Min. en.	123.8	137.4	156.4	164.2	13.6	26.8	40.4
338	Min. en.	79.7	97.6	112.3	117.8	17.9	20.2	38.1
348	Min. en.	132.2	146.2	168.0	177.6	14.0	31.3	45.3
358	Min. en.	156.5	175.3	200.5	211.9	18.8	36.5	55.3
368	Min. en.	246.5	261.9	295.3	304.9	15.4	42.9	58.4
278	Rand.	165.0	179.9	203.4	212.9	14.9	33.1	47.9
288	Rand.	203.2	217.7	243.4	252.6	14.4	34.9	49.4
298	Rand.	167.9	176.0	196.1	203.2	8.1	27.2	35.2
308	Rand.	123.7	138.7	158.3	166.6	15.0	27.9	42.9
318	Rand.	200.4	213.8	239.5	249.9	13.4	36.1	49.6
328	Rand.	134.3	150.5	173.2	182.3	16.2	31.8	48.0
338	Rand.	131.2	148.6	169.3	178.0	17.4	29.4	46.8
348	Rand.	155.7	170.3	191.0	197.9	14.6	27.6	42.3
358	Rand.	163.6	177.5	199.8	208.7	13.9	31.2	45.1
368	Rand.	97.2	105.7	120.1	125.8	8.6	20.0	28.6
Average		147.8	162.2	184.0	192.5	14.4	30.3	44.7
STD		39.7	39.9	44.0	45.1	3.0	5.9	7.0

[a] NR-TM: nonrelativistic Turbomole/Dalton-calculated shifts used for the snapshots.
[b] NR-ADF: nonrelativistic ADF.
[c] SR-ZO: scalar-relativistic (SR) part of the ZORA results.
[d] SO-ZO: both the SR and spin–orbit (SO) parts included at the ZORA level.
[e] Difference between NR-ADF and NR-TM.
[f] Difference between SO-ZO and NR-ADF.
[g] Difference between SO-ZO and NR-TM.

Table S6: Coefficients for the $Xe - H_2O$ pair potential (V_n) and chemical shift functions (δ_n) used in the NMR force field analysis of the MD snapshots (δ_n only) and the semianalytical cavity model (both V_n and δ_n), described in Section S1.8. PCS and EPCS stand for binary and effective pair chemical shift function, respectively

		$\delta_n(\mathrm{ppm} \cdot \mathrm{Å}^n)$	
n	$V_n(\mathrm{kcal/mol} \cdot \mathrm{Å}^n)$	PCS	EPCS
6	509813.157	-42300792.126	331491127
7	-14192000.4	1276710522.22	-10918170100
8	166263818	-16547206266.3	155848618000
9	-1074024180	120446322075.0	-1258224540000
10	4179852730	-539607787650.0	6282948000000
11	-10045391900	1527147215620	-19876027800000
12	14613195300	-2671464021010	38920341400000
13	-11816774200	2644810217460	-43157292300000
14	4083563200	-1135749395950	20761184000000

References

1 Werner, H.-J., Knowles, P. J., Knizia, G., Manby, F. R. & Schütz, M. Molpro: A general-purpose quantum chemistry program package. *WIREs Comput. Mol. Sci.* **2**, 242–253 (2012).

2 Werner, H.-J. *et al.* Molpro, version 2012.1, a package of ab initio programs (2012). See `http://www.molpro.net`.

3 Hampel, C., Peterson, K. A. & Werner, H.-J. A comparison of the efficiency and accuracy of the quadratic configuration interaction (QCISD), coupled cluster (CCSD), and Brueckner coupled cluster (BCCD) methods. *Chem. Phys. Lett.* **190**, 1–12 (1992).

4 Deegan, M. J. & Knowles, P. J. Perturbative corrections to account for triple excitations in closed and open shell coupled cluster theories. *Chem. Phys. Lett.* **227**, 321–326 (1994).

5 Peterson, K. A., Figgen, D., Goll, E., Stoll, H. & Dolg, M. Systematically convergent basis sets with relativistic pseudopotentials. ii. small-core pseudopotentials and correlation consistent basis sets for the post-*d* group 16–18 elements. *J. Chem. Phys.* **119**, 11113–11123 (2003).

6 Th. H. Dunning, Jr. Gaussian basis sets for use in correlated molecular calculations. I. The atoms boron through neon and hydrogen. *J. Chem. Phys.* **90**, 1007–1023 (1989).

7 Boys, S. F. & Bernardi, F. The calculation of small molecular interactions by the differences of separate total energies. some procedures with reduced errors. *Mol. Phys.* **19**, 553–566 (1970).

8 Frisch, M. J. *et al.* Gaussian 09 Revision D.01. Gaussian Inc. Wallingford CT 2009.

9 Norman, P., Schimmelpfennig, B., Ruud, K., H. J. Aa. Jensen & Ågren, H. Relativistic effects on linear and nonlinear polarizabilities studied by effective-core potential, Douglas–Kroll, and Dirac–Hartree–Fock response theory. *J. Chem. Phys.* **116**, 6914–6923 (2002).

10 Ren, P. & Ponder, J. W. Polarizable atomic multipole water model for molecular mechanics simulation. *J. Phys. Chem. B* **107**, 5933–5947 (2003).

11 Ren, P. & Ponder, J. W. Temperature and pressure dependence of the AMOEBA water model. *J. Phys. Chem. B* **108**, 13427–13437 (2004).

12 Kumar, A. & Meath, W. J. Integrated dipole oscillator strengths and dipole properties for Ne, Ar, Kr, Xe, HF, HCl, and HBr. *Can. J. Chem.* **63**, 1616–1630 (1985).

13 Mareš, J. & Vaara, J. Solvation structure and dynamics of Ni^{2+}(aq) from a polarizable force field. *Chem. Phys.* **443**, 112–122 (2014).

14 Ponder, J. W. *et al.* Current status of the AMOEBA polarizable force field. *J. Phys. Chem. B* **114**, 2549–2564 (2010).

15 Thole, B. Molecular polarizabilities calculated with a modified dipole interaction. *Chem. Phys.* **59**, 341–350 (1981).

16 Ren, P. & Ponder, J. W. Consistent treatment of inter- and intramolecular polarization in molecular mechanics calculations. *J. Comput. Chem.* **23**, 1497–1506

17 Allen, M. P. & Tildesley, D. J. *Computer simulation of liquids* (Clarendon Press, New York, 1989.).

18 Martyna, G. J., Tuckerman, M. E., Tobias, D. J. & Klein, M. L. Explicit reversible integrators for extended systems dynamics. *Mol. Phys.* **87**, 1117–1157 (1996).

19 Bussi, G., Zykova-Timan, T. & Parrinello, M. Isothermal-isobaric molecular dynamics using stochastic velocity rescaling. *J. Chem. Phys.* **130**, 074101 (2009).

20 Berendsen, H. J. C., Postma, J. P. M., van Gunsteren, W. F., DiNola, A. & Haak, J. R. Molecular dynamics with coupling to an external bath. *J. Chem. Phys.* **81**, 3684–3690 (1984).

21 Feller, S. E., Zhang, Y., Pastor, R. W. & Brooks, B. R. Constant pressure molecular dynamics simulation: The Langevin piston method. *J. Chem. Phys.* **103**, 4613–4621 (1995).

22 TURBOMOLE V6.5 2013, a development of University of Karlsruhe and Forschungszentrum Karlsruhe GmbH, 1989-2007, TURBOMOLE GmbH, since 2007; available from `http://www.turbomole.com`.

23 Angeli, C. *et al.* Dalton2011, a molecular electronic structure program (2011), see `http://www.daltonprogram.org`.

24 Becke, A. D. A new mixing of Hartree–Fock and local density-functional theories. *J. Chem. Phys.* **98**, 1372–1377 (1993).

25 Straka, M., Lantto, P. & Vaara, J. Toward calculations of the ^{129}Xe chemical shift in Xe@C_{60} at experimental conditions: relativity, correlation, and dynamics. *J. Phys. Chem. A* **112**, 2658–2668 (2008).

26 Lantto, P. & Vaara, J. ^{129}Xe chemical shift by the perturbational relativistic method: xenon fluorides. *J. Chem. Phys.* **127**, 084312 (2007).

27 Lantto, P., Standara, S., Riedel, S., Vaara, J. & Straka, M. Exploring new Xe-129 chemical shift ranges in HXeY compounds: hydrogen more relativistic than xenon. *Phys. Chem. Chem. Phys.* **14**, 10944–10952 (2012).

28 Eichkorn, K., Weigend, F., Treutler, O. & Ahlrichs, R. Auxiliary basis sets for main row atoms and transition metals and their use to approximate coulomb potentials. *Theor. Chem. Acc.* **97**, 119–124 (1997).

29 Vaara, J., Hanni, M. & Jokisaari, J. Nuclear spin-spin coupling in a van der waals-bonded system: Xenon dimer. *J. Chem. Phys.* **138**, 104313 (2013).

30 Roukala, J. *et al.* Encapsulation of xenon by a self-assembled Fe_4L_6 metallosupramolecular cage. *J. Am. Chem. Soc.* **137**, 2464–2467 (2015).

31 Hanni, M. *et al.* Electron and nuclear spin polarization in Rb-Xe spin-exchange optical hyperpolarization. *Phys. Rev. A* **95**, 032509 (2017).

32 E. van Lenthe, Baerends, E. J. & Snijders, J. G. Relativistic regular two-component Hamiltonians. *J. Chem. Phys.* **99**, 4597–4610 (1993).

33 E. van Lenthe, Baerends, E. J. & Snijders, J. G. Relativistic total energy using regular approximations. *J. Chem. Phys.* **101**, 9783–9792 (1994).

34 G. te Velde *et al.* Chemistry with ADF. *J. Comput. Chem.* **22**, 931–967 (2001).

35 ADF2016, SCM, Theoretical Chemistry, Vrije Universiteit, Amsterdam, The Netherlands, `http://www.scm.com`.

36 Schreckenbach, G. & Ziegler, T. Calculation of NMR shielding tensors using gauge-including atomic orbitals and modern density functional theory. *J. Phys. Chem.* **99**, 606–611 (1995).

37 Krykunov, M., Ziegler, T. & E. van Lenthe. Hybrid density functional calculations of nuclear magnetic shieldings using Slater-type orbitals and the zeroth-order regular approximation. *Int. J. Quantum Chem.* **109**, 1676–1683 (2009).

38 Autschbach, J. Magnitude of finite-nucleus-size effects in relativistic density functional computations of indirect NMR nuclear spin–spin coupling constants. *ChemPhysChem* **10**, 2274–2283 (2009).

39 E. van Lenthe & Baerends, E. J. Optimized Slater-type basis sets for the elements 1–118. *J. Comput. Chem.* **24**, 1142–1156 (2003).

40 Humphrey, W., Dalke, A. & Schulten, K. Vmd: Visual molecular dynamics. *J. Mol. Graphics* **14**, 33–38 (1996).

41 Friedberg, R. & Cameron, J. E. Test of the Monte Carlo method: fast simulation of a small Ising lattice. *J. Chem. Phys.* **52**, 6049–6058 (1970).
